# Improvement of cell freezing technologies: towards a fully closed process

**DOI:** 10.1186/1753-6561-5-S8-P1

**Published:** 2011-11-22

**Authors:** Aurore Polès-Lahille, Brigitte Lafuente, Virginie Perrier, David Balbuena, Didier Peyret

**Affiliations:** 1 Merck Serono Biodevelopment, Martillac, France, 33650

## 

Cell culture from vials usually includes an open phase performed under laminar flow hoods. Even if disposable and small closed systems are available, at least the first cell culture step after vial thawing, which is a centrifugation, is performed in disposable but open containers. This step is critical as the risk of contamination is high and it also has a direct impact on process timelines.

In order to reduce the risk of contamination during thawing, Merck Serono Biodevelopment studied the possibility of freezing cell banks in bags and to directly thawing them directly in disposable and closed containers.

The first step of this study was to evaluate if the DMSO present in freezing media had to be removed just after vial thawing or not. In general, this cryoprotective organic solvent is removed by centrifugation to avoid any toxic effect on cells.

So vials from two different mammalian cell lines were thawed, each with and without a centrifugation step to remove DMSO, or not. Afterwards the cell amplification was performed in the same way for the two different thawing procedures. The population doubling level and the viability were monitored for more than 15 days and there was no significant impact neither on cell growth nor on viability whether DMSO was removed or not just after thawing, or not. The important parameter to take into account was the percentage of DMSO after dilution which had to be less than 0,5%.

**Figure 1:**
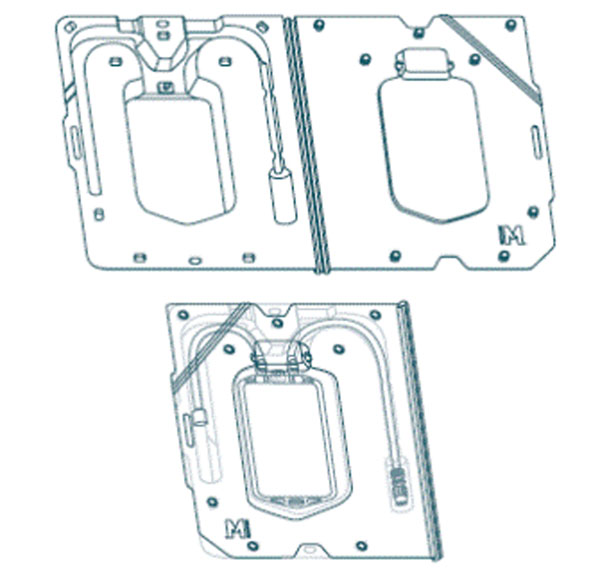
NovaSeptum 100mL bag and NovaSeptum case configurations

**Figure 2:**
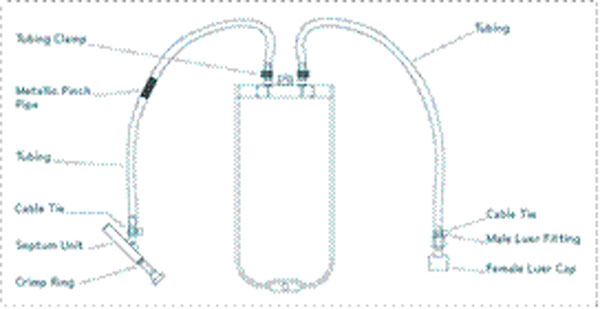
NovaSeptum 100mL bag and NovaSeptum case configurations

In order to reduce the risk of preparing cell banks, Merck Serono Biodevelopment looked at different disposable closed systems which allow cell banks to be prepared without laminar flow. It is possible to expand cells in closed containers such as spinners, shake flasks or rollers with deep tubes in addition to bags or disposable bioreactors. In order to concentrate cells or to change the medium from cell amplification to a cell freezing medium a centrifugation step may be necessary. Single use 500mL centrifuge tubes with deep tube and air vent are also available. In order to freeze cells, Merck Serono Biodevelopment evaluated several bags from several suppliers and chose the NovaSeptum^®^ 100mL bag and the NovaSeptum^®^ Case. The combination of these 2 elements ensured the integrity of the bag during freezing at -80°C (Figure 1-2).

Thus 50mL of two different concentrated mammalian cell suspensions were frozen at -80°C and thawed in disposable and closed containers. The cells were not centrifuged just after thawing but expanded in order to seed a production bioreactor. The cells were also maintained in order to see if there was a long term impact of non-DMSO removal on viability and cell growth. The population doubling level, the cell viability obtained in disposable closed cell culture containers in addition to viable cell density, metabolism, molecule quantity and quality obtained in bioreactors were monitored. These parameters were compared to the same process performed with cells coming from vials.

There was no process performance difference obtained between cells frozen in bags and cells frozen in vials. Cell freezing in bags without DMSO removal allows a fully closed cell culture and production process to be performed. It also allows process timelines to be reduced by least 2 weeks.

The study will continue with a focus on bags which are compatible with nitrogen containers. The quality aspect will also be studied in order to be able to prepare a full or part of manufacturing of cell banks in bags.

